# A 12-week evaluation of annatto tocotrienol supplementation for postmenopausal women: safety, quality of life, body composition, physical activity, and nutrient intake

**DOI:** 10.1186/s12906-018-2263-0

**Published:** 2018-06-28

**Authors:** Chwan-Li Shen, Shu Wang, Shengping Yang, Michael D. Tomison, Mehrnaz Abbasi, Lei Hao, Sheyenne Scott, Md Shahjalal Khan, Amanda W. Romero, Carol K. Felton, Huanbiao Mo

**Affiliations:** 1 Department of Pathology, Texas Tech University Health Sciences Center, Lubbock, Texas USA; 2 Laura W. Bush Institute for Women’s Health, Texas Tech University Health Sciences Center, Lubbock, Texas USA; 3 Department of Laboratory Sciences and Primary Care, Texas Tech University Health Sciences Center, Lubbock, Texas USA; 4 Department of Nutritional Sciences, Texas Tech University, Lubbock, TX USA; 5 Clinical Research Institute, Texas Tech University Health Sciences Center, Lubbock, Texas USA; 6 Department of Obstetrics and Gynecology, Texas Tech University Health Sciences Center, Lubbock, TX USA; 7 Department of Nutrition, Georgia State University, Atlanta, GA USA

**Keywords:** Vitamin E, Clinical trial, Dietary supplement, Liver function, Women, SF-36

## Abstract

**Background:**

Evidence suggests that tocotrienols may benefit bone health in osteopenic women. However, their safety in this population has never been investigated. This study was to evaluate the safety of a 12-week supplementation of annato tocotrienol in postmenopausal osteopenic women, along with effects of the supplementation on quality of life, body composition, physical activity, and nutrient intake in this population.

**Methods:**

Eighty nine postmenopausal osteopenic women were randomly assigned to 3 treatment arms: (1) Placebo (430 mg olive oil/day), (2) Low tocotrientol (Low TT) (430 mg tocotrienol/day from DeltaGold 70 containing 300 mg tocotrienol) and (3) High tocotrienol (High TT) (860 mg tocotrienol/day from DeltaGold 70 containing 600 mg tocotrienol) for 12 weeks. DeltaGold 70 is an extract from annatto seed with 70% tocotrienol consisting of 90% delta-tocotrienol and 10% gamma-tocotrienol. Safety was examined by assessing liver enzymes (aspartate aminotransferase, alanine aminotransferase), alkaline phosphatase, bilirubin, kidney function (blood urea nitrogen and creatinine), electrolytes, glucose, protein, albumin, and globulin at 0, 6, and 12 weeks. Serum tocotrienol and tocopherol concentrations were assessed and pills counted at 0, 6, and 12 weeks. Quality of life, body composition, physical activity, and dietary macro- and micro-nutrient intake were evaluated at 0 and 12 weeks. A mixed model of repeated measures ANOVA was applied for analysis.

**Results:**

Eighty seven subjects completed the study. Tocotrienol supplementation did not affect liver or kidney function parameters throughout the study. No adverse event due to treatments was reported by the participants. Tocotrienol supplementation for 6 weeks significantly increased serum delta-tocotrienol level and this high concentration was sustained to the end of study. There was no difference in serum delta-tocotrienol levels between the Low TT and the High TT groups. No effects of tocotrienol supplementation were observed on quality of life, body composition, physical activity, and nutrient intake.

**Conclusions:**

Annatto-derived tocotrienol up to 600 mg per day for 12 weeks appeared to be safe in postmenopausal osteopenic women, particularly in terms of liver and kidney functions. Tocotrienol supplementation for 12 weeks did not affect body composition, physical activity, quality of life, or intake of macro- and micro-nutrients in these subjects.

**Trial registration:**

ClinicalTrials.gov identifier: NCT02058420. Title: Tocotrienols and bone health of postmenopausal women.

## Background

Oxidative stress and low-grade inflammation have been considered to be the central mechanisms underlying the development of osteoporosis in postmenopausal women [1–3]. Recent studies demonstrate that nutritional supplements rich in antioxidants can mitigate the loss of bone matrix and deteriation of bone microstructure in the estrogen-deficient animal model, a model used to represent bone loss in postmenopaual women [4, 5].

Among different types of antioxidants, vitamin E is a collective term for tocotrienols and tocopherols. Tocotrienols and tocopherols each exist in four different forms in nature: alpha (α), beta (β), gamma (γ), and delta (δ) in mixtures of varying compositions [6]. Tocotrienol possesses an unsaturated sidechain, which afford more efficient penetration into cells compared with the completely saturated sidechain of tocopherols [7, 8]. Recently, tocotrienols, especially δ-tocotrienol, has gained an increasing interest due to its higher antioxidant-dependent biological activities in comparions with tocopherols [7, 8]. The order of anti-oxidant potency, as determined by ORAC values, is δ-tocotrienol > > γ-tocotrienol = β-tocotrienol = α-tocopherol > α-tocotrienol > γ-tocopherol = β-tocopherol > α-tocopherol [9, 10].

In a recent comprehensive review, the authors suggested that tocotrienols might reduce bone fracture risk by increasing bone mineral density and supporting osteoblastic activities while suppressing osteoclastic activities in preclinical studies [4]. The osteo-protective effects of tocotrienols are, in part, due to their antioxidant/anti-inflammatory functions [11] and suppressive effect on 3-hydroxy-3-methylglutaryl coenzyme A reductase [12].

Legislation in use of complementary and alternative medicine (i.e., herbal/dietary supplements) is inconsistent and even lacking in many countries. In the US, vitamin E is labeled as a dietary supplement that does not require pre-clinical tests because it is pre-Dietary Supplement Health and Education Act (DSHEA), or already in the market prior to the enactment of the DSHEA of 1994. Most of the published tocotrienol clinical safety studies were either short-term (≤ 2 weeks) [13, 14] or using a mixture of tocotrienol and tocopherol [15, 16]. With a longer study period, only single low dose of tocotrienol was used [16]. A study with high dose tocotrienol had limited safety data related to liver and kidney functions [17]. Furthermore, there is limited clinical information on the safety of δ-tocotrienol supplementation in humans [13, 17, 18]. The limited number of published δ-tocotrienol studies on pharmacokinetics and bioavaiability was either relatively short-term or with a small sample size, and they were not randomized placebo-controlled trial [13, 17, 18]. The detailed safety information at a higher dosage is increasingly important because of mounting interest in clinical studies using tocotrienols. Lacking such safety information hinders research development. Therefore, the present work was the first annatto seed-extract tocotrienol safety report on liver and kidney functions based on two different dosages (300 and 600 mg tocotrienol) with a larger sample size in a 12-week double-blinded placebo-controlled randomized clinical trial. The objective of this paper was to evaluate the safety of tocotrienol supplementation for 12 weeks in postmenopausal osteopenic women. In addition to safety, the effects of treatment arms on quality of life, as assessed by Short Form-36 (SF-36) questionnaires, serum tocotrienol and tocopherol concentrations (as assessed by high pressure liquid chromatography), body composition (as assessed by composition analyzer), physical activity (as assessed by Godin Leisure-Time Exercise Questionaire), and nutrientintake (as assessed by food frequency questionnaires) were also reported.

## Methods

### Study design and intervention

This was a 12-week, double-blinded, placebo-controlled, randomized intervention trial to investigate the effects of tocotrienol on bone parameters. Participants were randomly assigned to one of the three treatment groups: Placebo group (0 mg tocotrienol/day, 430 mg olive oil), Low tocotrienol (Low TT) group (430 mg DeltaGold 70 containing 300 mg tocotrienol/day), and High tocotrienol (High TT) group (860 mg DeltaGold 70 containing 600 mg tocotrienol/day).

Placebo and tocotrienol supplementof the same lot were supplied by American River Nutrition, Inc., Hadley, MA (US Food and Drug Aministation, Investigational New Drug (IND) number 120,761). Placebo softgels were made of the same size and color as the tocotrienol softgelts for identifical appearance and taste. DeltaGold 70, an extract from annatto seed with 70% purity, consisted of 90% delta-tocotrienol and 10% gamma-tocotrienol. During the 12-week intervention, all participants were provided with 500 mg elemental calcium and 200 IU vitamin D (as cholecalciferol) daily. Trial registration: ClinicalTrials.gov identifier: NCT02058420. Title: Tocotrienols and bone health of postmenopausal women.

### Study participants

The complete study protocol was reported in detail previously [19] and only a brief description is provided here. Inclusion criteria were (i) postmenopausal women (at least 1 years after menopause) age 45 and older with osteopenia (mean hip and/or lumbar spine bone mineral density T-score between 1 and 2.5 standard deviation (SD) below the young normal sex-matched areal bone mineral density of the reference database) [12], (ii) normal function of thyroid, liver, and kidney, (iii) serum 25-hydroxy vitamin D ≥ 20 ng/mL, and (iv) no bisphosphonate treatment at least 12 months before study began. Women were excluded if they (i) had a disease condition or were on medication known to affect bone metabolism; (ii) had a history of cancer within the last 5 years; (iii) had hormone/hormone-like replacement therapy within 6 months of the study initation; (iv) had endocrine disease, malabsorption syndrome, cognitive impairment, depression, or other medical/eating disorders; (v) had a history of statin or other cholesterol-reducing drugs within 3 months of the study initiation; (vi) smoked > 10 cigarettes/day, had alcohol intake > 1 drink/day or used non-steroidal anti-inflammatory drugs on a regular basis; (vii) had HbA1c > 7.0; and (viii) were unwilling to accept randomization. Written informed consent was obtained from all the study participants before enrollment. The study was approved by the Texas Tech University Health Sciences Center Institutional Review Board.

### Randomization and blinding

To ensure comparable distribution across treatment arms, stratified randomization was applied to eligible participants to balance baseline covariates, including age (≥ 50 or < 50 yr) and body mass index (BMI) (≥ 30 or < 30 kg/m^2^). The study participants and investigators were blinded to the group allocation.

### Compliance and adverse event monitoring

Adherence/compliance of tocotrienol or placebo study agents was determined as the percentage of all of tocotrienol or placebo softgels ingested throughout the study period. In the course of the 12-week clinical trial, adverse events associated with tocotrienol were self-reported by the participants, and by monitoring liver enzyme activities, aspartate aminotransferase (AST) and alanine aminotransferase (ALT) in particular, through blood analysis. All observed and self-reported adverse events, regardless of suspected causal relationship to the study treatments, were recorded on the adverse event form throughout the study.

### Bioavailablity: Serum vitamin E concentration

To evaluate the bioavailability of study agents, the concentations of serum tocotrienol and tocopherol at baseline, 6, and 12 weeks were determined using a high-pressure liquid chromatography (HPLC) system based on our previously published method [19]. Briefly, serum was saponified in 10% KOH solution containing 0.001% of butylated hydroxytoluene acid and 1.1% of ascorbic acid at 95 °C for 30 mintues. *Rac*-Tocol was used as an internal standard. After saponification, vitamin E was extracted using hexane, and dried under a nitrogen evaporator. Tocotrienols and tocopherols were detected using a Waters HPLC system equipped with a silica column (5 μm, 4.6 × 250 mm) and a florescence detector. The mobile phase was composed of hexane and 1–4 dioxane (96:4, volume:volume), and the flow rate was 2.0 mL/min. The excitation and emission wavelengths were 296 nm and 325 nm, respectively.

### Data collection and outcome measures

Body composition was measured at baseline and every 6 weeks via bioimpedance measurement (SC-331S Body Composition Analyzer, Tanita Corporation of America, Inc., Arlington Height, IL, USA). Physical activity level, food intake, and quality of life were collected at the baseline and after 12 weeks. Physical activity level was assessed via Godin Leisure-Time Exercise Questionaire. Food nutritent intake was assessed by a semiquantitative Harvard Willett Food Frquency Questionnaire. Quality of life status was assessed with the Medical Outcomes Study 36-item short form Health Survey (SF-36, version 2), which consists of eight dimensions of health (physical function, bodily pain, general health, vitality, mental health, social function, and role of physical and emotional health) in the conduct of daily activity [20].

Laboratory comprehensive metabolic panel including liver function (AST, ALT, bilirubin, and alkaline phosphatase activity (ALP)), kidney function (blood urea nitrogen (BUN), and creatinine), electrocytes (calcium (Ca), sodium (Na), potassium (K), chloride (Cl)), carbon dioxide (CO_2_), and others (glucose, total protein, albumin, and globulin) were assessed in overnight fasting blood samples taken at baseline, 6, and 12 weeks. All samples were processed and analyzed in a certified diagnostic laboratory (Quest Diagnostic Laboratory, Dallas, TX).

### Statistical analysis

We used power analysis and sample size software (PASS11) to obtain a sample size of 22 in each of the 3 arms (Placebo, Low TT, and High TT) for a power of 80% in our bone biomarker study [11]. Such a sample size was able to detect a clinically significant difference between the group means of urine N-terminal telopeptide (NTX, primary outcome measure in the main study) level at the end of 12 weeks [19]. This calculation is based on an analysis of covariance (ANCOVA), adjusted for three covariates: baseline NTX, age, and body mass index (BMI). With an expected attrition rate of 15%, a total of 78 participants were recruited (*n* = 26 for each group) to start the study.

An “intent-to-treat” principle was adopted in the data analysis. Descriptive statistics were used to describe the characteristics of the study cohort. Categorical variables were summarized as frequencies, and continuous variables were summarized using mean and standard deviation. Participant characteristics were compared to detect differences among the three groups at baseline. Also at each time point, Chi-squared test/Fisher’s exact test or one way analysis of variance was used to compare whether there was any difference among the three groups, as appropriate. To compare the differences in change over time among the three groups, a repeated measure ANOVA was used controlling within-subject correlation. Statistical software SAS 9.4 (Cary, NC, USA) was used for all the analyses. A *p* value less than 0.05 was considered statistically significant.

## Results

### Participants

A total of 416 participants were prescreened. Among them, 89 were qualified and randomized, and 87 completed the 12-week study. Two participants (1 in the Placebo group and 1 in the Low TT group) withdrew before the end of the study, both due to loss of interest. Baseline characteristics were similar among different treatment groups (Table 1). All subjects were instructed to maintain their pre-existing physical activity, dietary habits, and medications, if any, throughout the study. Based on the results of pill count, the compliance rates were 92.9, 91.7, and 90.5% for the Placbo group, Low TT group, and High TT group respectively, showing no significant difference among the groups.

**Table 1:** Baseline demographic characteristics of study population

Variables	Placebo	Low TT	High TT	*P* value
Number	28	29	30	
Age [y]	59.4±6.3	58.5±6.7	61.2±7.2	0.431
Weight [kg]	74.3±17.9	76.3±14.8	74.3±16.8	0.680
Height [cm]	160.0±6.2	163.7±6.5	155.6±27.1	0.107
Body mass index [kg/m^2^]	28.9±6.5	28.5±5.0	28.8±5.8	0.986
Bone mineral density [T-score]				
Femoral Neck	−1.66±0.65	−1.52±0.82	−1.34±1.17	0.795
Trochanter	−1.06±0.95	−1.04±0.79	−0.85±1.30	0.945
Total spine	0.79±0.89	−0.87±0.85	−0.53±1.10	0.654
L1-L4	−0.67±0.99	−0.72±1.17	0.59±1.23	0.919
Serum 25 (OH)D [ng/mL]	35.79±9.13	35.45±9.70	34.27±9.97	0.634
Serum TSH [mIU/L]	2.25±1.01	2.45±1.46	2.52±1.71	0.944
Serum HbA1c [%]	5.66±0.35	5.81±0.34	5.56±0.36	0.011
Medical history [n (%)]				
Broken bone as adult	6 (21.43)	7 (24.14)	8 (26.67)	0.897
Osteoarthritis	3 (12.00)	5 (20.83)	3 (11.54)	0.584
Diabetes	2 (7.14)	2 (6.90)	0 (0.00)	0.331
Asthma	4 (14.29)	3 (10.34)	2 (6.67)	0.636
Use of HRT in the past 2 yr	10 (35.71)	8 (27.59)	6 (20.00)	0.409
Steroid or glucocorticoid use	5 (17.86)	4 (13.79)	3 (10.00)	0.687
Prescribed osteoporosis drugs	1 (3.57)	4 (13.79)	7 (23.33)	0.093

All data are mean ± standard deviation unless otherwise specified

*HbA1c* hemoglobin A1c, *High TT* tocotrienol supplementation at 860 mg daily (70% purity); *HRT* hormone replacement treatment, *Low TT* tocotrienol supplementation at 430 mg daily (70% purity), *TSH* thyroid stimulating hormone, *25(OH)D* 25-hydroxy-vitamin D

### Serum tocotrienol and tocopherol concentrations

Table 2 shows the effects of tocotrienol supplementation on blood concentrations of tocotrienol and tocopherol. At the baseline, there was no significant difference in any forms of tocotrienol and tocopherol among the three treatments. Based on the results of repeated ANOVA controlling within-subject correlation, significant difference in the overtime change of serum δ-tocotrienol was found among the three groups (*p* = 0.042). Compared with the Placebo group, a significant overtime increase in serum δ-tocotrienol concentration was found in Low TT and High TT groups (*p* = 0.017 and *p* = 0.048, respectively).

**Table 2:** Concentrations (μmol/L) of tocopherols and tocotrienols in various treatment groups at weeks 0, 6 and 12

VE	Week 0			Week 6			Week 12			Overall P Value
	Placebo^a^	Low TT	High TT	Placebo	Low TT	High TT	Placebo	Low TT	High TT	
α-tocopherol	14.64 (6.89)	14.3 (5.71)	12.38 (6.35)	14.03 (7.4)	13.56 (4.43)	13.67 (5.39)	16.17 (6.44)	15.01 (5.64)	12.42 (6.31)	0.771
β-tocopherol	1.86 (1.86)	3.21 (3.70)	2.72 (2.26)	3.07 (2.49)	2.26 (2.19)	2.50 (1.92)	2.43 (2.35)	2.82 (2.66)	2.57 (2.04)	0.571
δ-tocopherol	1.17 (1.22)	1.59 (2.52)	0.94 (1.31)	1.44 (2.41)	1.64 (2.26)	1.98 (1.43)	1.43 (2.37)	2.38 (3.23)	1.84 (1.25)	0.649
γ-tocopherol	2.58 (2.71)	3.79 (3.32)	3.18 (2.15)	3.13 (2.75)	4.38 (3.13)	3.27 (1.83)	3.79 (3.61)	4.23 (3.49)	2.57 (2.07)	0.189
α-tocotrienol	0.92 (1.69)	1.73 (3.45)	1.82 (2.23)	2.68 (3.73)	1.89 (2.81)	0.96 (1.25)	0.87 (1.84)	1.20 (1.93)	1.79 (2.27)	0.847
β-tocotrienol	0.34 (0.70)	0.11 (0.44)	0.49 (0.97)	0.61 (0.87)	0.59 (0.91)	0.33 (0.61)	0.20 (0.54)	0.12 (0.31)	0.91 (1.40)	0.341
δ-tocotrienol	0.54 (0.92)	1.06 (2.34)	0.77 (1.08)	1.05 (2.39)	1.93 (1.81)	2.32 (1.33)	1.14 (1.84)	3.19 (2.71)	2.59 (2.10)	0.042
γ-tocotrienol	0.40 (0.84)	0.77 (2.61)	1.04 (2.56)	1.58 (4.83)	1.46 (2.99)	1.27 (2.09)	1.08 (2.65)	2.71 (4.25)	1.74 (3.24)	0.307

^a^Values are mean(SD). Repeat measures ANOVA was used to evaluate whether the slopes of change in VE concentration overtime were the same for the three interventions

### Blood chemistry profiles

Table 3 depicts the effects of tocotrienol supplementation on blood chemistry at baseline, 6, and 12 weeks. At the baseline, there was no significant difference in any of the blood chemistry parameters among all treatment groups. Based on the results of repeated ANOVA, the levels of serum AST, ALT, and bilirubin (indicators of liver function) were not affected by tocotrienol intervention during the 12-week study period. Similarly, tocotrienol supplementation did not influence kidney function (BUN and creatinine), electrolytes (Ca, Na, K, Cl, CO_2_), and any other blood biochemistry parameters (glucose, protein, albumin, and globulin) in the study participants throughout the study period.

**Table 3:** Effect of tocotrienol supplementation on blood parameters in postmenopausal osteopenic women

Variable	Week 0			Week 6			Week 12			Overall P Value
	Placebo^a^	Low TT	High TT	Placebo	Low TT	High TT	Placebo	Low TT	High TT	
AST (U/L)	23.01 (10.68)	20.76 (6.57)	20.77 (4.95)	21.46 (5.53)	20.76 (6.76)	22.07 (7.97)	21 (5.23)	20.21 (9.39)	21.9 (7.63)	0.515
ALT (U/L)	23.75 (11.49)	19.9 (8.35)	19.03 (7.83)	20.82 (8.77)	19.66 (8.88)	20.5 (10.34)	21.14 (8.67)	18.72 (8.65)	19.57 (10.76)	0.494
Bilirubin (mg/dL)	0.53 (0.19)	0.52 (0.14)	0.57 (0.15)	0.46 (0.21)	0.48 (0.13)	0.43 (0.11)	0.5 (0.19)	0.47 (0.15)	0.47 (0.12)	0.583
ALP (U/L)	72.75 (14.68)	79.59 (15.53)	79.23 (22.96)	74.04 (16.95)	76.28 (18.04)	75.23 (16.56)	74.64 (15.6)	75.14 (16.31)	74.4 (16.99)	0.730
BUN (mg/dL)	14.46 (3.48)	16.41 (4.6)	16.57 (4.26)	14.86 (3.33)	15.62 (3.53)	17.07 (4.18)	15.54 (3.96)	16.97 (3.74)	16.67 (4.44)	0.120
Creatinine (mg/dL)	0.79 (0.12)	0.85 (0.13)	0.83 (0.12)	0.82 (0.12)	0.87 (0.15)	0.86 (0.15)	0.82 (0.13)	0.89 (0.17)	0.83 (0.12)	0.111
Ca (mg/dL)	9.62 (0.38)	9.54 (0.37)	9.48 (0.29)	9.44 (0.35)	9.41 (0.36)	9.37 (0.39)	9.57 (0.34)	9.55 (0.53)	9.5 (0.28)	0.607
Na (mmol/L)	138.86 (3.43)	139.72 (1.56)	139.27 (2.33)	139.5 (2.85)	140.03 (2.5)	140.1 (2.17)	139.75 (1.9)	140.48 (2.35)	140.37 (2.51)	0.346
K (mmol/L)	3.99 (0.28)	4.03 (0.36)	4.02 (0.35)	3.83 (0.24)	3.77 (0.29)	3.78 (0.4)	3.84 (0.32)	3.91 (0.31)	3.79 (0.39)	0.986
Cl (mmol/L)	103.71 (3.55)	104.1 (2.55)	104.1 (2.92)	103.61 (2.81)	103.55 (2.32)	104.5 (1.87)	103.29 (2.68)	103.9 (3.02)	104.13 (3.04)	0.314
CO_2_ (mmol/L)	21.68 (2.28)	22.48 (2.29)	22.87 (2.61)	19.79 (2.22)	21.1 (1.97)	20.83 (2.35)	20.89 (1.83)	21.45 (1.74)	20.67 (2.92)	0.086
Glucose (mg/dL)	90.61 (10.78)	88.21 (13.91)	85.8 (15.05)	87.43 (11.81)	83.34 (12.29)	83.27 (14.1)	84.75 (11.78)	83.83 (12.74)	83.83 (11.75)	0.282
Protein, total (g/dL)	7.19 (0.41)	7.23 (0.48)	7.17 (0.54)	7.2 (0.37)	7.24 (0.4)	7.2 (0.45)	7.29 (0.33)	7.24 (0.44)	7.31 (0.41)	0.810
Albumin (g/dL)	4.39 (0.26)	4.36 (0.21)	4.35 (0.26)	4.32 (0.24)	4.28 (0.2)	4.26 (0.18)	4.41 (0.19)	4.33 (0.21)	4.38 (0.19)	0.474
Globulin (g/dL)	2.8 (0.35)	2.87 (0.41)	2.82 (0.45)	2.89 (0.3)	2.97 (0.41)	2.95 (0.39)	2.87 (0.28)	2.91 (0.37)	2.94 (0.38)	0.468
Albumin/globulin	1.59 (0.22)	1.55 (0.25)	1.59 (0.26)	1.52 (0.19)	1.48 (0.23)	1.46 (0.21)	1.55 (0.16)	1.52 (0.2)	1.52 (0.22)	0.451

^a^Values are mean(SD). *AST* asparate aminotransferase, *ALT* alanine aminotransferase, *ALP* alkaline phosphatase, *BUN* blood urea nitrogen, *Ca* calcium, *Cl* chloride, *CO*_*2*_ carbon dioxide, *K* potassium, *Na* sodium

### Quality of life

Data demonstrating the effects of tocotrienol supplementation on quality of life, including all 8 domains, in postmenopausal osteopenic women are presented in Table 4. There were no significant differences in any domain of quality of life among the Placebo, the Low TT, and the High TT groups at the baseline, 6 weeks, and 12 weeks (*p* > 0.05). Futhermore, throughout the course of the 12-week intervention, there was no statistically significant change in any domain with time in all treatment groups (*p* > 0.05).

**Table 4:** Effect of tocotrienol supplementation on quality of life in postmenopausal osteopenic women

Domain	Week 0			Week 12			Overall P Value
	Placebo^a^	Low TT	High TT	Placebo	Low TT	High TT	
Physical function	89.64 (12.24)	83.1 (16.71)	88 (13.68)	77.22 (10.13)	69.29 (16.82)	74.66 (10.93)	0.779
Role-physical	92.19 (11.98)	87.5 (18.3)	89.79 (15.09)	93.75 (11.63)	84.15 (21)	92.71 (12.07)	0.293
Bodily pain	71.43 (16.04)	68.28 (19.65)	71 (13.98)	72.22 (15.77)	71.07 (19.5)	71 (16.26)	0.761
General health	80.18 (16.03)	78.07 (14.7)	83.5 (15.86)	80.56 (17.42)	78.57 (15.76)	83.67 (14.78)	0.754
Vitality	66.29 (16.52)	63.58 (19.7)	66.46 (17.71)	65.97 (16.84)	67.63 (20.2)	73.54 (15.8)	0.079
Social function	94.2 (15.02)	92.24 (14.72)	91.67 (16.52)	93.06 (15.24)	89.29 (23)	94.58 (12.14)	0.167
Role-emotional	89.29 (19.62)	92.24 (14.07)	93.89 (10.48)	90.74 (13.54)	91.67 (16.67)	93.61 (9.71)	0.818
Mental health	80.71 (12.82)	84.31 (12.44)	80.17 (15.51)	82.04 (16.42)	84.82 (14.37)	83.33 (13.22)	0.637

^a^Values are mean(SD)

### Body composition and physical activity

There was no significant difference between baseline and 12 weeks in body weight, % body fat, and BMI among the three groups, and the corresponding *p* values were 0.574, 0.733, and 0.449, respectively (Table 5). At baseline, the Placebo group had more exercise sessions than those in the High TT group, while there was no difference in estimated exercise time among the Placebo, Low TT, and Hight TT groups. There was no significant difference between baseline and at 12 weeks in physical activity in terms of exercise frequency and exercise time among the three groups (Table 5).

**Table 5:** Effect of tocotrienol supplementation on body composition and physical activity in postmenopausal osteopenic women

Variable	Week 0			Week 12			Overall P Value
	Placebo^a^	Low TT	High TT	Placebo	Low TT	High TT	
Body Composition							
BMI (kg/m^2^)	28.95 (6.47)	28.47 (4.98)	28.77 (5.78)	27.88 (6.44)	28.37 (5.28)	28.95 (6.01)	0.345
Fat (%)	39.13 (7.4)	39.41 (6.89)	39.01 (7.48)	38.97 (7.22)	39.81 (7.28)	39.37 (7.59)	0.959
Weight (kg)	74.26 (17.94)	76.34 (14.76)	74.33 (16.76)	72.87 (16.99)	75.56 (15.25)	74.36 (16.72)	0.504
Physical activity profile							
Exercise frequency (sessions/week)	2.14^a^ (0.85)	1.96^ab^ (0.88)	1.60^b^ (0.62)	2.19 (0.62)	2.0 (0.86)	1.75 (0.80)	0.869
Exercise time (min/session)	30.26 (22.53)	31.07 (27.36)	34.30(19.08)	34.74(23.32)	29.69(26.45)	39.83(25.93)	0.318

Within a given row, values that share the same superscript letter (a, b) are not statistically different from each other after adjusting for age and BMI

^a^Values are mean(SD)

### Nutrient intake

Table 6 presents the comparison of the before (0 week) and after (12 weeks) study difference in nutrient intake among the treatments. At baseline, there was no statistical difference in any macro- and micro-nutrients intake among three treatments. As expected, throughout the 12-week study period, no difference in macro- and micro-nutrients intake in study groups was observed (*p* > 0.05) (Table 6).

**Table 6:** Effect of tocotrienol supplementation on macro- and micro-nutrient intake in postmenopausal osteopenic women

Variable	Week 0			Week 12			Overall P Value
	Placebo^a^	Low TT	High TT	Placebo	Low TT	High TT	
Total calories (kcal)	1685.9 (624.72)	2399.8 (1549.82)	1928.2 (810.37)	1610.4 (550.15)	2128.6 (1382.71)	2211.9 (1816.89)	0.111
Carbohydrates (g)	199.8 (86.99)	266.5 (169.59)	232.5 (106.84)	190.2 (73.18)	232.9 (159.45)	264.0 (241.36)	0.233
Total sugars (g)	90.7 (52.64)	123.6 (77.6)	112.8 (60.48)	87.2 (42.48)	103.3 (68.72)	124.1 (126.57)	0.192
Total fiber (g)	21.7 (8.99)	27.8(19.94)	24.1 (10.73)	20.7 (8.67)	26.7 (19.18)	29.4 (26.49)	0.252
Total fat (g)	65.9 (26.97)	104.4 (71.73)	72.7 (33.8)	63.3 (24.00)	95.4 (61.70)	84.5 (62.57)	0.215
Cholesterol (mg)	204.5 (99.95)	323.7 (245.7)	297.2 (190.79)	212.5 (95.05)	268.0 (196.27)	309.8 (256.2)	0.278
Saturated fat (g)	20.6 (10.61)	33.5 (25.52)	25.1 (15.02)	19.7 (8.13)	28.0 (18.52)	26.6 (21.59)	0.213
Monounsaturated (g)	25.0 (10.02)	40.2(27.25)	25.90 (11.60)	24.1 (9.04)	38.7 (25.83)	31.8 (21.11)	0.201
Polyunsaturated (g)	14.2 (6.27)	20.9 (14.32)	14.6 (6.66)	13.7 (7.14)	20.2 (14.56)	18.3 (15.28)	0.298
Protein (g)	72.4 (27.83)	101.2 (71.44)	90.0(41.12)	70.5 (23.93)	91.2(74.78)	105.0(97.63)	0.293
Calcium (mg)	1164.1 (587.92)	1277.4 (730.38)	1318.2(792.1)	1184.6 (517.16)	1044.3 (574.77)	1219.5 (1044.16)	0.373
Potassium (mg)	2908.1 (1107.18)	3892.8 (2582.54)	3343.0(1560.48)	2847.1 (972.19)	3463.7 (2495.8)	3970.2 (3551.94)	0.202
Phosphorus (mg)	1211.7 (486.11)	1638.9 (1057.27)	1480.8(655.18)	1217.3 (408.83)	1430.9 (1027.86)	1655.4 (1476.94)	0.309
Sodium (mg)	1849.3 (826.99)	2656 (1860.41)	2337.2(963.73)	1847.2 (719.41)	2355.3 (1609.08)	2609.3 (2047.47)	0.257
Magnesium (mg)	382.1 (183.15)	490.1 (331.28)	413.0(223.5)	363.5 (148.08)	405.1 (273.53)	446.2 (437.75)	0.396
Iron (mg)	12.2 (4.64)	17.2 (11.74)	16.0 (9.24)	12.4 (7.81)	15.5 (12.2)	18.2 (14.12)	0.435
α-Carotene (μg)	703.2 (548.58)	1143.1 (2215.95)	619.2 (475.97)	713.0 (585.51)	1180.0 (2996.32)	1402.5 (2523.89)	0.133
β-Carotene (μg)	5288.3 (3804.98)	6993.7 (9886.87)	5531.6 (3336.1)	5379.9 (4162.6)	7229.7 (11,253.44)	7927.5 (10,896.58)	0.439
Vitamin D (IU)	437.0 (400.57)	443.7 (296.05)	516.0 (403.89)	412.5 (339.19)	286.4 (256.39)	382.7 (371.33)	0.333
Vitamin E (mg)	16.9 (35.42)	29.7 (70.48)	17.2 (19.49)	18.5 (34.65)	61.2 (80.46)	26.3 (46.57)	0.365
Vitamin K1 (μg)	174.1 (132.15)	217.9 (173.9)	228.8 (133.54)	178.5 (130.91)	214.1 (179.3)	253.0 (231.18)	0.805
Vitamin C (mg)	266.4 (351.41)	224.5 (266.92)	163.4 (211.03)	300.2 (410.03)	159.3 (226.95)	209.8 (242.44)	0.058
Thiamine (mg)	3.6 (9.69)	6.5 (14.13)	5.4 (13.11)	5.5 (13.72)	3.5 (10.24)	5.3 (12.86)	0.762
Riboflavin (mg)	4.2 (9.75)	7.2 (14.11)	6.2 (13.35)	6.1 (13.56)	4.0 (10.37)	6.2 (13.17)	0.744
Pyridoxine (mg)	8.1 (29.29)	7.1 (14.19)	13.0 (34.1)	8.0 (16.3)	17.9 (39.61)	11.5 (38.01)	0.255
Vitamin B12 (μg)	46.9 (146.1)	51.5 (137.43)	46.9 (127.98)	70.3 (168.15)	66.1 (168.15)	11.8 (14.73)	0.138
Folic acid (μg)	153.7 (130.66)	277.0 (263.85)	234.0 (301.46)	215.1 (235.85)	161.1 (203.88)	205.6 (191.8)	0.121
Niacin (mg)	25.6 (11.66)	49.7 (57.12)	34.2 (22.63)	47.2 (78.42)	30.3 (25.59)	37.0 (32.63)	0.186

^a^Values are mean(SD)

## Discussion

This was the first double-blinded placebo-controlled randomized study to evaluate the safety of 12-week ingestion of annatto seed-extracted tocotrienol in postmenopausal women. This study demonstrated that tocotrienol supplementation of up to 600 mg tocotrienol daily for 12 weeks did not cause any safety concerns with regard to liver function (in terms of AST, ALT, ALP, and bilirubin levels), kidney function (in terms of BUN and creatinine levels), or other blood chemistry parameters. In fact, tocotrienol supplementation seemed to improve liver function by showing reduced ALP in the tocotrienol-treated groups. The findings that tocotrienol supplementation had no adverse effects on liver function is in agreement with a study conducted by Magosso et al. [16]. In Magosso’s study, one-year supplementation of mixed tocotrienol isomers (α-tocotrienol, δ-tocotrienol and γ-tocotrienol) and α-tocopherol at 200 mg twice daily did not change blood levels of ALT, AST, glucose, or creatinine in patients with nonalcoholic fatty liver disease [16].

In the present study, we reported that tocotrienol supplementation for 12 weeks did not affect body composition in terms of weight, BMI, and %body fat of study participants. These findings were consistent with Magosso et al. [16], who showed that 1 year of mixed tocotrienol and tocopherol supplementation did not change BMI in patients with nonalcoholic fatty liver disease.

Quality of life is affected by many factors, including physical, social and emotional functions that are in turn modulated by certain chronic conditions such as pain and inflammation. Accumulating evidence suggests the link between mitochondrial dysfunction, oxidative stress and neuropathic pain that could be treated with antioxidants [21]. Oxidative stress and neuroinflammation are among the mechanisms underlying neurodegenerative diseases, adversely affecting mental health and quality of life [22]. This study also evaluated the effect of TT supplementation on the quality of life, and the results showed no adverse effect. There was no evidence supporting that vitamin E (tocopherols) intake benefited the quality of life in patients with amyothrophic lateral sclerosis [23]. Another study found that green tea extract supplementation, also rich in antioxidants, for 24 weeks did not benefit the quality of life in postmenopausal osteopenic women in a randomized placebo-controlled intervention [24]. Although all these supplements (TT, tocopherols, and green tea polyphenols) are considered to be functional in protecting cells from oxidative stress, these published studies along with the present study seem to suggest no impact of these supplements on the quality of life. As expected, tocotrienol supplementation did not influence physical activity or nutrition intake in subjects at 12 weeks (end of study), since the subjects were advised to maintain the same lifestyle. It may be prudent to refrain from interpreting the lack of impact of tocotrienol supplementation on physical activity or nutrition intake until further studies since the subjects were advised to maintain the same lifestyle.

In the present study, we measured the concentrations of 4 isomers of tocotrienol and 4 isomers of tocopherol in the overnight-fasting serum of subjects, and we found that only serum δ-tocotrienol concentrations were elevated in the tocotrienol-supplemented groups at 6 weeks and sustained at 12 weeks. The serum δ-tocotrienol concentrations in the tocotrienol-supplemented group reflected the composition of tocotrienol supplement consisting of 90% δ-tocotrienol + 10% γ-tocotrienol. Due to the small amount of γ-tocotrienol in the study supplement, we were not able to observe the difference in serum γ-tocotrienol levels among the three treatment groups. There was neither dose-effect (300 mg in the Low TT group vs. 600 mg in the High TT group) nor time-effect (6 weeks vs. 12 weeks) in tocotrienol-supplemented groups. In addition, we also found serum β-tocotrienol was elevated in tocotrienol-adminstered groups, although the concentration of β-tocotrienol was relative low. Qureshi et al. recently reported the safety and pharmacokinetics of single doses of 750 mg/day and 1000 mg/day of annatto-based tocotrienol, which was the same study agent used in our study, in healthy humans (30–40 year, *n* = 3 per group) by collecting plasma at 0, 1, 2, 4, 6, and 8 h intervals after meal [17]. The authors found (1) the higher single dose of tocotrienol was safe in humans; (2) Tmax was 3–4 h for all isomers of tocotrienol and tocopherol except for α-tocopherol at 6 h; and (3) the plasma levels and areas under curve of all tocotrienol isomers, δ-tocopherol, and β-tocopherol were affected in a dose-dependent fashion. Based on the finidngs of Qureshi et al. [17], it is possible to observe the elevated plasma levels of all tocotrienol isomers, including β-tocotrienol, after supplementation of annatto-based tocotrienol. The reasons why we were not able to observe a dose- and time-dependent manner in our study were (i) because the tocotrienol had cleared the entero-hepatic circulation after 6–8 h, whereas blood draw occurred at 12 h after an overnight fast, and (ii) saturation of tocotrienol [25] after a longer period of intervention (e.g., 6 weeks).

As expected, TT supplementation for 6 weeks significantly increased serum delta-TT levels and this high concentration was sustained to the end of study with continued supplementation. Although the supplement (DeltaGold) is Generally Recognized as Safe (GRAS), it is still worthy to include a follow-up assessment of serum vitamin E concentration and safety in a future study.

## Conclusion

Supplementation of 600 mg tocotrienol daily to postmenopausal osteopenic women for 12 weeks did not cause any adverse effects on liver and kidney function, as determined by blood test parameters, and had no influence on quality of life (as assessed by SF-36 questionnaires), body composition, physical activity, and nutritent intake. Based on our findings, tocotrienol at a dose of 600 mg per day for 12 weeks appears to be safe in postmenopausal osteopenic women.
